# How many (distinguishable) classes can we identify in single-particle analysis?

**DOI:** 10.1107/S2059798325007831

**Published:** 2025-09-09

**Authors:** O. Lauzirika, M. Pernica, D. Herreros, E. Ramírez-Aportela, J. Krieger, M. Gragera, M. Iceta, P. Conesa, Y. Fonseca, J. Jiménez, J. Filipovic, J. M. Carazo, C. O. S. Sorzano

**Affiliations:** a Centro Nacional de Biotecnologia–CSIC, Calle Darwin 3, 28049Cantoblanco, Madrid, Spain; b Faculty of Informatics, Masaryk University, Brno, Czech Republic; c Institute of Computer Science, Masaryk University, Brno, Czech Republic; National Centre for Biological Sciences-TIFR, India

**Keywords:** cryo-electron microscopy, 3D classification, structural heterogeneity, statistical significance, reproducibility analysis

## Abstract

Heterogeneity in cryoEM is essential for capturing the structural variability of macromolecules, reflecting their functional states and biological significance. However, estimating heterogeneity remains challenging due to particle mis­classification and algorithmic biases, which can lead to reconstructions that blend distinct conformations or fail to resolve subtle differences. Furthermore, the low signal-to-noise ratio inherent in cryo-EM data makes it nearly impossible to detect minute structural changes, as noise often obscures subtle variations in macromolecular projections. In this paper, we investigate the use of *p*-values associated with the null hypothesis that the observed classification differs from a random partition of the input data set, thereby providing a statistical framework for determining the number of distinguishable classes present in a given data set.

## Abstract 2

Estimating structural heterogeneity in cryo-EM remains difficult due to noise, misclassification and algorithmic biases that obscure subtle conformational differences. This work introduces a statistical framework based on *p*-values to assess whether a given classification reflects meaningful structural variation or is indistinguishable from random partitioning.

## 1. Introduction

The presence of structural heterogeneity in cryo-EM data sets is not merely a source of noise but also a unique opportunity to probe the conformational landscape of macromolecular machines. By capturing particles in multiple states, cryo-EM can reveal distinct conformations that correspond to functionally relevant intermediates, providing valuable insights into the underlying energy landscape. At the same time, this heterogeneity imposes significant demands on image-analysis methods: algorithms must disentangle genuine structural variability from the overwhelming background noise, while avoiding artificial splitting of homogeneous populations. This dual role of heterogeneity, as both a window into biological function and a source of computational complexity, highlights the need for principled approaches to determine the number of structurally meaningful classes reliably.

Classification approaches can be broadly categorized as discrete or continuous (Sorzano *et al.*, 2019 ▸; Tang *et al.*, 2023 ▸; Toader *et al.*, 2023 ▸; Kimanius & Schwab, 2024 ▸). Discrete classification aims to group particles into distinct, non-overlapping classes, each representing a specific structural conformation of the macromolecule. This method is ideal for resolving well separated states, such as open and closed conformations, but struggles with more nuanced structural variability or when the number of distinct states is unclear. On the other hand, continuous classification models structural variability as a smooth continuum of conformations, better capturing gradual transitions or flexible regions within the macromolecule. In some cases, continuous methods can achieve a clear separation of conformations in the latent space, as illustrated in the CryoBench study (Fig. 6 in Jeon *et al.*, 2024 ▸). Nevertheless, many other approaches within this family, especially under low signal-to-noise conditions, still encounter substantial challenges, including high computational demands and difficulties in result interpretation. Ultimately, the ability to identify discrete classes depends both on the data set and on the specific method employed.

This paper focuses on discrete classification, designed to address cases of true discrete heterogeneity and approximate continuous heterogeneity by quantization. As highlighted in Sorzano *et al.* (2022 ▸), discrete classification can be highly unstable, especially in noisy data sets. Factors such as class imbalance, the ‘3D attraction effect’ and inconsistencies across repeated classifications often compromise the accurate identification of structural classes. A central reason behind the instability of discrete 3D classification lies in the misestimation of the parameters that define the partitioning process. Such misestimation arises from two main factors: firstly, the intrinsic nondeterministic nature of classification algorithms, which typically start their optimization from different random initial conditions and may therefore converge to distinct local solutions; and secondly, the extremely low signal-to-noise ratio (SNR) of cryo-EM images, which produces a highly rugged optimization landscape with numerous local minima. These factors jointly increase the likelihood that repeated runs of the same algorithm yield divergent classifications, even when the data contain meaningful structural heterogeneity. These challenges underscore the importance of developing robust methodologies to enhance classification reliability and call for the cautious interpretation of results. In this context, this paper examines the limitations of current approaches and explores potential strategies for improvement.

In Sorzano *et al.* (2022 ▸), we proposed that a practical way to identify parameter misestimates is to estimate the same parameter multiple times, preferably using different algorithms or, at the very least, through various executions of the same algorithm with nondeterministic variations, such as random initialization. For discrete 3D classification, this approach entails performing the classification repeatedly at each level. While this practice is rarely adopted due to its high computational cost, the demonstrated instability of 3D classification underscores the risk of basing biological interpretations solely on the results of a single execution, potentially leading to compromised conclusions.

Several works have addressed the problem of reliably estimating the number of classes and/or their composition:

(i) Rabuck-Gibbons *et al.* (2022 ▸) tackled the instability of 3D classification in cryo-EM data sets by introducing a workflow that iteratively divides an input data set into two smaller subsets until the volume difference between the subclasses falls below a predefined threshold. Subsequently, similar maps are merged into larger classes through hierarchical clustering using their correlation as a similarity measure. However, this approach presents two limitations: determining an appropriate signal threshold for stopping the subdivision process is challenging, and the user must select the final number of clusters based on the dendrogram, which can be difficult to interpret, especially for complex data sets.
(ii) In a similar vein, Gomez-Blanco *et al.* (2022 ▸) proposed a divisive hierarchical approach where, at each step, the data set is split into two or three classes. The stopping criteria for further subdivision were: (i) the number of particles in a class must exceed a user-defined threshold (typically 5000–10 000 particles) and (ii) the resolution of the class, as determined by the classification method, must surpass the resolution predicted by the ResLog plot for the given particle count. If both conditions were satisfied, the class was further divided. Finally, to group similar maps, each map was represented by a feature vector derived from its truncated PCA representation, and clustering was performed using the affinity-propagation algorithm, which measured distances in the feature vector space using the Euclidean metric.
(iii) Zhou *et al.* (2022 ▸) tackled the problem by incrementally classifying the input data set into *K* = 2, 3, 4, … classes. They measured the similarity between raw particle images and their corresponding reconstructed 3D references for each classification. The optimal number of classes was determined as the *K* that maximized the average variance of this similarity score within each class. The rationale is that an incorrect number of classes, either too few or too many, reduces the within-class variance. With too few classes, particles from different structural states are mixed, making it impossible for the representative model to accurately reflect any subset of particles. With too many classes, noise overfitting occurs, artificially increasing the particle similarity within each class. As highlighted by Tibshirani *et al.* (2001 ▸), selecting the number of classes solely by inspecting within-cluster dispersion, such as minimizing intra-class variability, can be misleading and prone to substantial error, as it lacks a principled statistical basis. This concern is well recognized in the pattern-recognition community, prompting the development of more statistically grounded approaches, such as the gap statistic.

A significant limitation of all of these approaches is their reliance on a single classification step for each *K*, which inherently makes the results susceptible to the stochastic nature and instability of the classification process, particularly as *K* (the number of classes) increases. In single-particle analysis, classification instability arises due to noise, particle misalignments and biases introduced by initial conditions or by the specific algorithm. As *K* grows, separating particles into increasingly finer classes amplifies the risk of overfitting noise or misassigning particles to incorrect classes. This can result in artificial or noisy classes, blending of distinct conformations or failure to identify low-populated conformational states.

An alternative strategy for estimating the number of classes, without resorting to explicit classification, is to analyze the rank of the 3D covariance matrix of the particle images. In principle, the number of distinct classes should be at least the rank plus one (Katsevich *et al.*, 2015 ▸; Andén & Singer, 2018 ▸). Modern methods, such as *RECOVAR* (Gilles & Singer, 2025 ▸), enable the direct estimation of the 3D covariance matrix from noisy cryo-EM images, thereby bypassing the need for prior classification. However, in practice, the very low signal-to-noise ratio typical of cryo-EM severely complicates the identification of a clear spectral gap in the eigenvalue distribution, making it difficult to determine the number of significant principal components and, consequently, the actual number of classes.

In this article, we propose a divisive approach similar to the methods introduced by Rabuck-Gibbons *et al.* (2022 ▸) and Gomez-Blanco *et al.* (2022 ▸). Our approach uses binary subdivisions, as dividing into more classes can increase classification instability. However, unlike previous methods, we repeat the same subdivision multiple times to estimate the consistency. The stopping criterion is determined by the *p*-value associated with the null hypothesis that the observed subdivision is random rather than reflecting the genuine structural features of the images. This statistical foundation ensures that the divisions are driven by meaningful data characteristics rather than noise or arbitrary thresholds on the signal difference, the number of particles or the class resolution. We are aware that performing multiple executions of the same classification step may be computationally tedious; however, at present, it remains the only practical way to estimate the uncertainty of the classification outcome. In the future, developers may introduce faster classification algorithms or alternative techniques to quantify classification uncertainty more efficiently.

## 2. Methods

**Figure 1:**
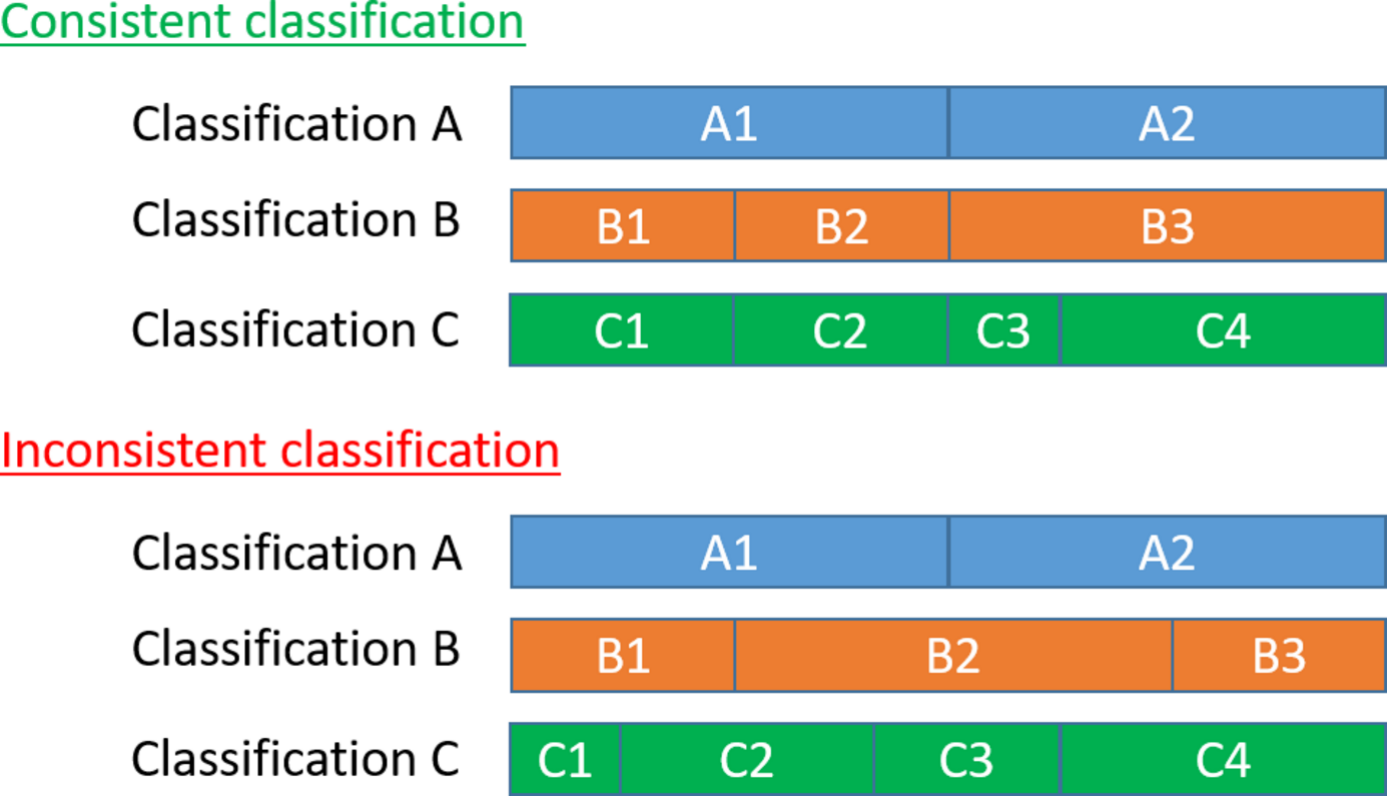
Examples of consistent and inconsistent classifications of a set of images into two, three and four classes, named A, B and C. In consistent classification the grouping structure of the larger groups is respected, whereas in inconsistent classification it is not.

Suppose we partition a set of *N* images into *K* classes and that the images truly exhibit a discrete class structure. In that case, we expect them to be consistently classified into stable groups, regardless of the number of classes used for the split, as illustrated in Fig. 1 ▸. If the classification algorithm successfully identifies the underlying classes, specific subsets of images should consistently remain grouped across different levels of the hierarchy. For instance, in the provided example, we would expect the images in Class 1 of Classification C to also be grouped in Class B1 and Class A1 at higher levels of the hierarchy. Conversely, if the classification algorithm fails to separate the images consistently, we would expect the images to be randomly distributed among the classes in subsequent classifications. This will be our null hypothesis. In this scenario, let 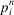 represent the proportion of images in the *i*th class during the *n*th classification. The expected proportion of images classified into Class *i*_1_ in the first classification, *i*_2_ in the second and so on, up to *i*_*n*_ in the *n*th classification, would be given by 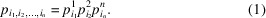


The expected size of this set can be modeled using a binomial distribution with parameters *N* and 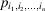. However, in our classification, which we aim to ensure is not random, we observe an actual size 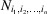. To assess whether this observed size is consistent with the null hypothesis of random classification, we calculate the probability of observing a size equal to or greater than 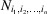 under the null hypothesis. This probability provides a *p*-value associated with each observed size. We reject the null hypothesis of random classification if this size is smaller than a given threshold (typically 0.05, although we may lower it to 0.01 or even smaller; in all our experiments we used 0.05).

It is essential to note that the reproducibility of a classification, as assessed by our method, does not guarantee its accuracy in itself. A classification can appear stable across repetitions, not because it reflects the true structural heterogeneity of the data set, but due to a persistent bias introduced by the underlying algorithm, its initialization, regularization strategies or the weighting of specific image features. To address this limitation, the most robust application of our framework involves comparing classifications obtained from different algorithms. By incorporating cross-method comparisons into the core of the procedure, we aim to reduce the risk of algorithm-specific biases being mistaken for true structural features. While our statistical framework quantifies the internal consistency of the classification process, it does not detect systematic biases that may be shared across repeated runs of the same method. In this sense, the technique evaluates classification variability, not correctness. Complementary validation steps, such as inspecting the resulting 3D reconstructions, performing cross-method comparisons or interpreting the results in light of prior biological knowledge, remain critical for assessing the plausibility and validity of the identified classes.

In our divisive algorithm, each input data set is split into two classes. This process is repeated *n* times to identify subsets of images that are consistently and significantly grouped, as described above. These identified subsets are treated as new classes, which can then be further divided into smaller subsets. Images that cannot be consistently separated are combined into a new class and reintroduced into the algorithm. The algorithm continues branching until no further meaningful divisions can be made. At this point, that branch is terminated. While our choice of binary subdivision is arbitrary, the method is general and would apply equally to splits into three or more classes. However, as the number of output classes increases, the classification typically becomes less stable, making it harder to identify statistically significant and reproducible groupings. Therefore, binary splits provide a practical balance between expressive power and robustness.

Our divisive algorithm is flexible and independent of the specific classification algorithm used to separate the images. Several options can be employed: (i) a classification algorithm that uses previously estimated angular assignments, keeping them fixed while estimating only the class memberships, or (ii) a joint estimation of angular assignments and class memberships, which can be performed either as a local refinement (using prior estimates) or as a global refinement (without relying on prior estimates).

## 3. Results

We validated our algorithm on several representative data sets, utilizing a classification workflow that incorporated multiple steps as necessary. These steps might include map reconstruction using Fourier gridding techniques (Abrishami *et al.*, 2015 ▸), local refinement of angular and translational parameters with *Xmipp highres* (Sorzano *et al.*, 2018 ▸) and unsupervised 3D classification in *cryoSPARC* without alignment.

### 3.1. Apoferritin

Apoferritin is a standard benchmark in cryo-electron microscopy (cryoEM) due to its structural stability and is frequently used for calibration purposes. Given its homogeneity, we did not expect our method to identify multiple classes. We analyzed 17 000 polished particles from EMPIAR-11796 (Aiyer *et al.*, 2024 ▸), using their *RELION*-derived pose assignments. The original authors reconstructed the structure under an imposed octahedral symmetry constraint, and in our study we used the same set of angular assignments. The particles were classified into two groups across three independent *cryoSPARC* runs without alignment. As anticipated, the resampled classifications consistently resembled random partitions, and the statistical test correctly failed to reject the null hypothesis (*p*-value = 0.45).

### 3.2. Ribosembly

**Figure 2:**
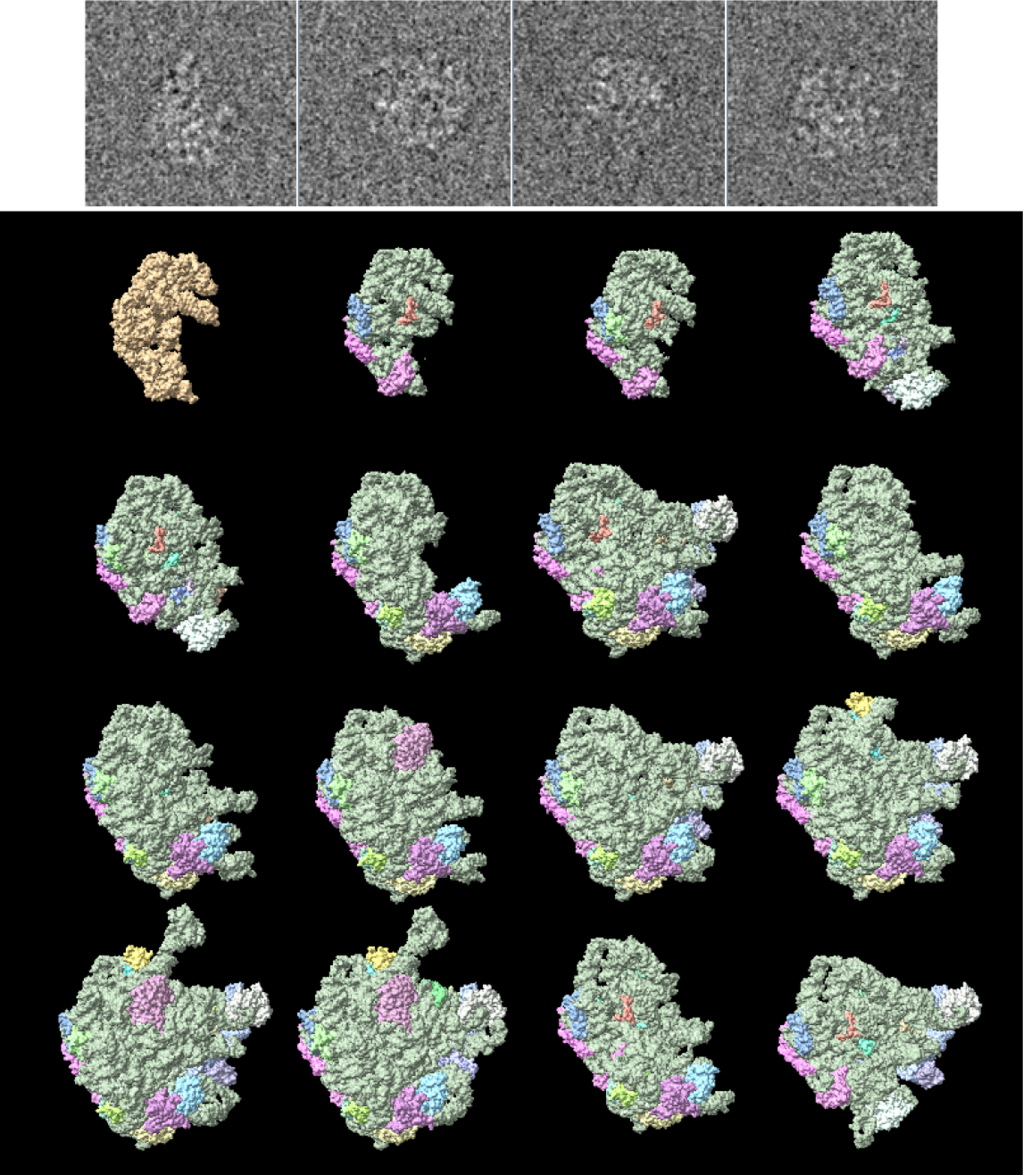
Some simulated projections and the 16 structures present in the Ribosembly data set from CryoBench.

The Ribosembly data set, part of the CryoBench benchmark suite (Jeon *et al.*, 2024 ▸), contains 335 240 synthetic cryo-EM particle images corresponding to 16 well defined bacterial ribosome assembly intermediates. These intermediates share a conserved structural core and progressively incorporate additional proteins and rRNA, providing a realistic model of compositional heterogeneity during ribosome maturation. The data set includes ground-truth atomic structures, density maps and imaging parameters, making it well suited for assessing the performance of classification algorithms. Fig. 2 ▸ presents a selection of particle projections together with the 16 ground-truth structures, providing the reader with a visual impression of the complexity of the classification problem.

We applied our divisive classification workflow iteratively until no further statistically significant subdivisions could be detected. Starting from the known angular assignments, each iteration consisted of reconstructing the candidate subgroups with *RELION*’s Fourier gridding approach and then performing three independent two-class classifications in *cryoSPARC* without alignment. To assess reproducibility, we compared the three resulting partitions: particles that were consistently assigned to the same class across all runs were merged to define a consensus class, which we interpret as a stable grouping. For branches where the subdivisions were inconsistent across runs, we treated the corresponding non­significant subsets as mixtures; these particles were pooled back together and reintroduced into the workflow for further rounds of binary splitting. This process was repeated until no new statistically significant divisions were obtained.

Table 1 ▸ shows the distribution of particle images across the 16 ground-truth classes in the Ribosembly data set and their assignments after two iterations of our classification procedure. Although the workflow involved additional iterations, we limit the table to the first two levels for clarity. The results show that some ground-truth classes are relatively well preserved across classifications; for example, most particles from classes 3 and 14 consistently remain grouped. In contrast, other classes, such as 0 and 7, appear more dispersed. While this is not a strict rule, it is generally observed that large ground-truth classes tend to remain cohesive. In contrast, smaller classes are more prone to fragmentation across output classes. It is essential to note that our algorithm does not introduce this dispersion; rather, it reflects the behavior of the underlying 3D classification method. Our algorithm groups particles that were consistently assigned together by the 3D classifier.

We continued the splitting procedure until no further statistically significant divisions could be made, ultimately resulting in 113 classes. Although the underlying ground truth comprises only 16 classes, the 3D classification algorithm failed to distinguish them accurately from the outset. For example, the actual class 0 became dispersed across multiple second-iteration classes (C11, C12, C13, C14, C21 and C22, as shown in Table 1 ▸). Consequently, additional splits were required to resolve the mixtures introduced by these initial misclassifications. The resulting 113 classes had an average purity of 0.93 (as defined in the legend of Table 1 ▸), with class sizes ranging from 41 486 to 145 particles. This demonstrates that our splitting procedure does not merely fragment homogeneous groups into smaller subsets; instead, it applies a statistical criterion for subdivision, independent of class size. For comparison, a single *cryoSPARC* classification into 16 classes yielded a much lower average purity of 0.50.

**Figure 3:**
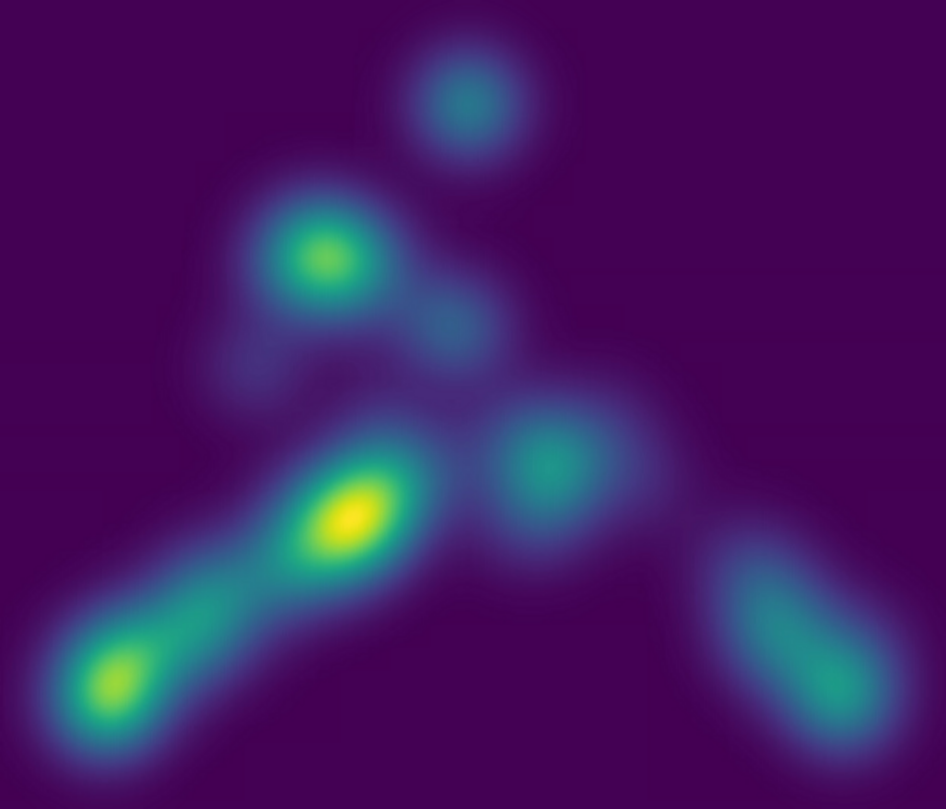
*StructMap* projection of the Ribosembly data set. The projection is a two-dimensional latent space obtained by applying multidimensional scaling (MDS) to the pairwise distance matrix of the reconstructed maps. In this space, the axes have no direct physical meaning. Still, they are chosen to best preserve the relative similarities: maps that are structurally more similar are placed closer together, while more distinct maps are placed farther apart. The pseudocolor represents the local density of maps in the projection, with brighter regions corresponding to positions where multiple maps overlap.

Fig. 3 ▸ displays the *StructMap* projection (Sorzano *et al.*, 2016 ▸) of the resulting maps onto a two-dimensional plane. As expected, the projection reveals distinct clusters without smooth transitions, consistent with discrete heterogeneity. Apparent overlaps between some classes are projection artifacts resulting from reducing the original 113 × 113 distance matrix to two dimensions.

We acknowledge that our procedure can generate more classes than the ground-truth number, effectively oversplitting some conformations. However, this effect is alleviated by the *StructMap* analysis, which groups closely related maps together and reveals that many of the oversplit classes correspond to local subdivisions of the same structural state. By contrast, the *cryoSPARC* classification into 16 classes achieved the correct number only because it was imposed *a priori*, and the resulting groups showed extensive mixing, leading to a much lower average purity. This highlights that our method prioritizes the recovery of reproducible, unmixed classes, even at the cost of temporary oversplitting, which can subsequently be consolidated through *post-hoc* analysis of class similarity.

### 3.3. bL17-limited *Escherichia coli* 50S ribosome

The EMPIAR-10841 data set has traditionally been used as a benchmark for discrete 3D classification (Rabuck-Gibbons *et al.*, 2022 ▸). The data set comprises cryo-electron microscopy (cryo-EM) raw movies and processed particle stacks of bL17-limited *E. coli* 50S ribosomal subunit assembly intermediates. This data set was generated to investigate the structural heterogeneity inherent in ribosome assembly, particularly under conditions where the ribosomal protein bL17 is depleted. This example corresponds to an experimental equivalent of the ribosome assembly simulated data set.

**Figure 4:**
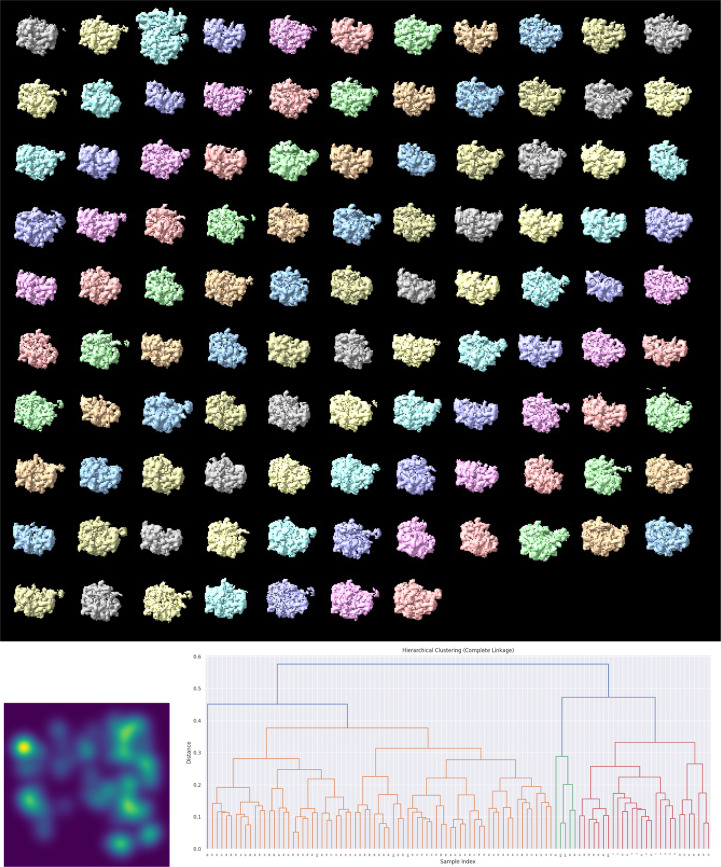
Classification of the bL17-limited *E. coli* 50S ribosome data set. Top: 3D reconstructions of the particles assigned to each of the classes. Bottom: *StructMap* projection of the maps and dendrogram representation of their hierarchical clustering. In the dendrogram, maps that are more similar are joined earlier (closer to the leaves), while those that are more dissimilar merge later (closer to the root). The color code is used solely to aid visualization, with groups defined by a default threshold set at 0.7 × max(distance).

**Figure 5:**
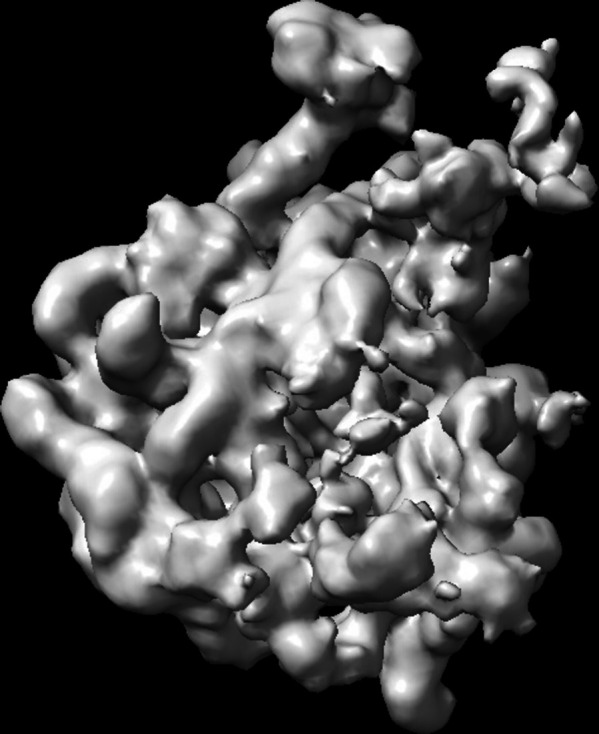
Example reconstruction of the smallest class of the ribosome data set.

We initiated our 3D classification using the aligned particles from the data set. Each iteration involved three independent *cryoSPARC* classifications, the identification of statistically distinguishable classes, Fourier reconstruction of each class using *RELION*’s gridding method and local angular refinement with *Xmipp highres*. The original study reported the presence of 41 classes, but since the assignments of individual particles to these classes were not made available in EMPIAR, a direct comparison with our results is not possible. Using our approach, we identified 106 statistically distinguishable classes (see Fig. 4 ▸), with particle counts ranging from 400 (see Fig. 5 ▸) to 3400 and an average of approximately 1300 particles per class. These classes capture both compositional and conformational variability present in the data set.

### 3.4. AftD from mycobacteria, mutant R1389S

The EMPIAR-10391 data set contains particle images and angular assignments for two conformational states of arabinofuranosyltransferase from a mutant *Mycobacterium* strain (Tan *et al.*, 2020 ▸). According to the original study, the first state comprises 37 814 particles and the second state includes 68 231. We attempted unsupervised 3D classification into two classes using both *RELION* and *cryoSPARC*, each repeated three times. Without masking, neither software could reproducibly separate the two conformations. To improve separation, we applied a spherical mask focused on the region of greatest difference between the two corresponding EMDB maps (EMD-21600 and EMD-21601).

Using *cryoSPARC* for focused classification within the masked region, we repeated the analysis three times and obtained three statistically distinct outcomes: (i) 55 631 particles split 1.5%/98.5%, (ii) 46 822 particles split 78%/22% and (iii) 3592 particles split 13.6%/86.4%. These proportions remained consistent across repetitions, suggesting that the original classification likely included approximately 20% of Class 1 images that were erroneously assigned to Class 2. To better resolve this heterogeneity, we applied our classification algorithm without angular refinement, justified by the small spatial region distinguishing the classes, and identified 49 statistically distinct classes, with particle counts ranging from 5369 to 518.

**Figure 6:**
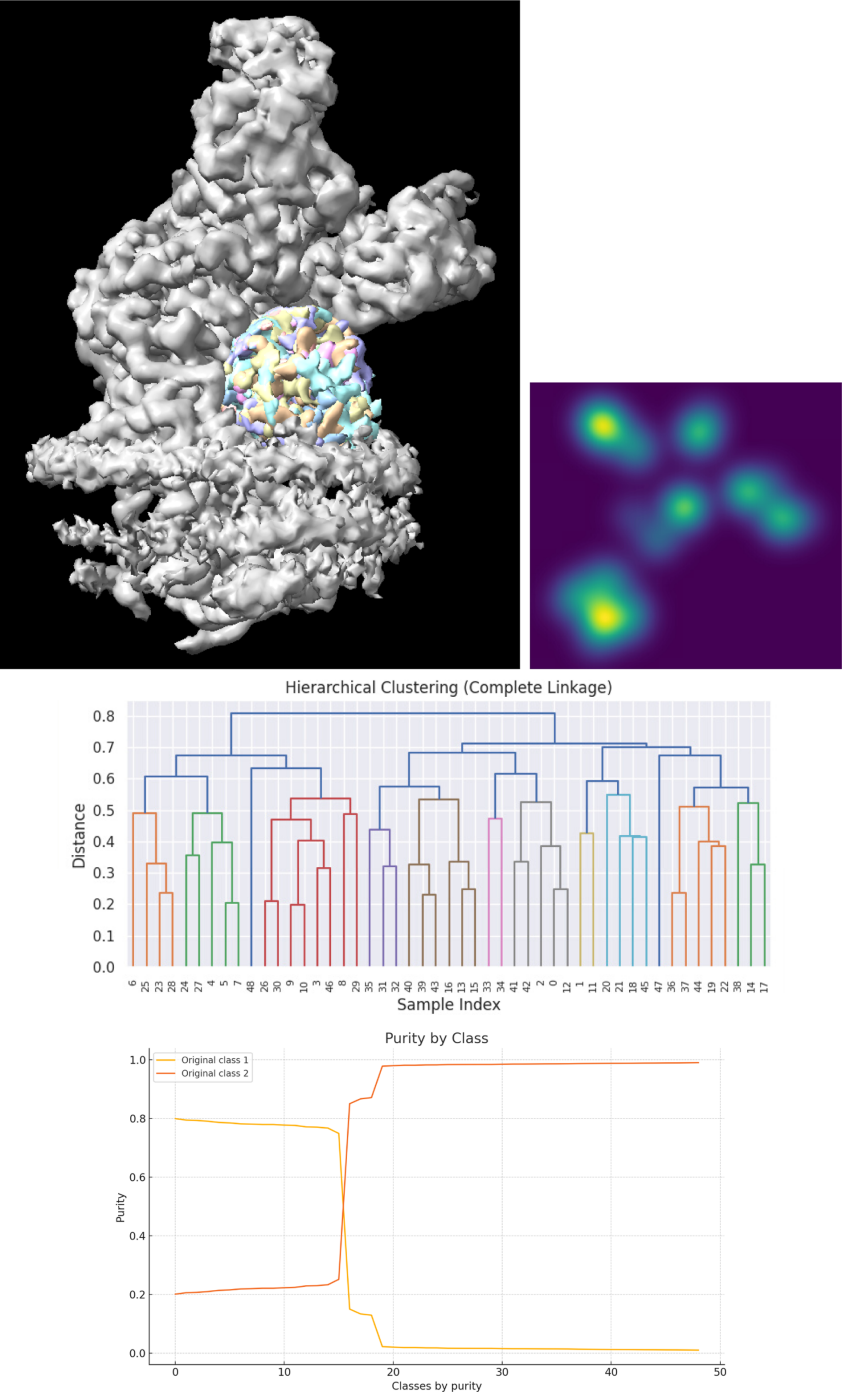
Top left: *ChimeraX* visualization of the 49 classes detected in the EMPIAR-10391 data set. Top right: *StructMap* representation of the 49 classes in a two-dimensional projection (the brightness corresponds to multiple classes projecting onto the same coordinate). Middle: dendrogram representation of the hierarchical clustering of the maps using complete linkage. Bottom: purity of each of the 49 calculated classes in terms of the original classification deposited in EMPIAR.

As illustrated in Fig. 6 ▸, the structural variability within this region is substantial. The *StructMap* projection displays all classes in a two-dimensional space, revealing both densely populated clusters and clearly separated regions. These results demonstrate the ability of standard discrete 3D classification to distinguish structurally distinct particle populations.

While the original two-class scheme captured some of this heterogeneity (as shown in the dendrogram in Fig. 6 ▸), it oversimplified the landscape. Many of our classes align closely with the original Class 2, while others correspond mainly to Class 1, although some include particles initially misclassified as Class 2. Apart from this 20% contamination, the original classification underused the potential of discrete classification to uncover the full structural diversity present in the data set.

## 4. Discussion

Determining the number of meaningful classes in single-particle cryo-EM data sets remains a fundamental and unresolved challenge, particularly in the presence of compositional or conformational heterogeneity. Existing approaches often rely on heuristic criteria, such as class size, map resolution or visual assessment of class similarity, to guide the classification process. However, such thresholds are inherently arbitrary, lacking a principled statistical foundation, and may lead to inconsistent or nonreproducible results across data sets and users.

In this work, we introduce a novel strategy that incorporates a statistically rigorous criterion, *p*-values derived from hypothesis testing, to determine whether a given classification step reflects a meaningful structural distinction or merely arises from random partitioning. This framework provides a technically sound basis for deciding when to accept or reject a proposed subdivision, independent of particle count, estimated resolution or user-defined parameters. By grounding the classification decision in statistical significance, our approach helps to mitigate algorithmic bias (when multiple algorithms are used for classification) and subjectivity in interpreting classification results. It is essential to emphasize that the assumption of randomness applies only to the null hypothesis, which we aim to disprove. We do not assume that the classification is random; in fact, we think the opposite. By placing the random classification scenario in the null hypothesis, we can use *p*-values to test whether the observed groupings are statistically distinguishable from randomness, thereby validating their significance. It is essential to clarify that by formulating the null hypothesis as random classification, we do not imply that classification algorithms typically behave in a completely erratic manner. Instead, randomness here refers to situations where the observed subdivisions are indistinguishable from those that would be expected if particles were assigned arbitrarily; for instance, when the data set lacks true discrete classes (as in the case of apoferritin) or when the signal of structural differences is buried under noise. Even when genuine heterogeneity is present, the inherent stochasticity of current algorithms can cause borderline particles to switch classes across runs, producing results that partially resemble random partitions. Our statistical framework is designed precisely to detect and evaluate these cases, distinguishing between reproducible structural features and subdivisions that could arise by chance.

Although some algorithms, such as *RELION* (Scheres, 2012 ▸), internally compute posterior probabilities of particle assignment to each class, their outputs are ultimately reduced to hard partitions, and the full probability distributions are not readily accessible to the user; consequently, our statistical framework, which is built on hard assignments, cannot directly exploit this additional information.

A critical advantage of our method is that it does not inherently favor the formation of small classes. On the contrary, we have demonstrated its flexibility across a broad spectrum of class sizes: in some data sets, statistically distinguishable classes contained several tens of thousands of particles, while in others valid classes were composed of just a few hundred. This adaptability underscores the data-driven nature of our approach, which bases subdivisions on reproducibility rather than on fixed size or resolution thresholds.

Our results also highlight the intrinsic instability of current discrete 3D classification algorithms. Even when using the same input data and classification parameters, repeated runs can yield divergent class assignments, a consequence of noise, alignment uncertainties and algorithmic nondeterminism. Rather than avoiding this variability, our method embraces it by explicitly testing the reproducibility of classifications. Subsets of particles that consistently appear together across independent runs are considered to be statistically significant, while unstable groupings are rejected. In this way, the instability of 3D classification becomes a measurable property that can be harnessed to improve confidence in the final set of classes.

Despite its advantages, our algorithm is ultimately constrained by the capabilities of the underlying 3D classification algorithm used to detect structural differences. The statistical framework that we propose relies on the reproducibility of these classifications; however, if the 3D classifier fails to resolve an actual structural distinction, due, for example, to a low signal-to-noise ratio (SNR) or subtle conformational differences, then no statistical test can recover that information. In this sense, the sensitivity of our method is fundamentally limited by the detectability of differences in the data itself. When structural variations fall below the resolution threshold imposed by noise or insufficient particle count, even repeated classification and hypothesis testing may fail to identify meaningful subdivisions. Thus, while our algorithm adds robustness to the interpretation of 3D classification results, it cannot compensate for the inherent limitations of the data or the algorithms used to process it.

Our results on the synthetic data set illustrate an essential property of the method: in the presence of substantial conformational and compositional diversity, the procedure may yield more classes than the number of distinct states present in the ground truth. We view this tendency as a consequence of the algorithm prioritizing sensitivity to heterogeneity. In practice, this means that an additional post-analysis step is often required to consolidate highly similar classes into a smaller number of biologically interpretable states. This is consistent with previous work, such as that of Rabuck-Gibbons *et al.* (2022 ▸), who initially obtained 30 classes from the bL17-limited *E. coli* 50S ribosome data set and subsequently grouped them into 12 representative structures based on a minimum mass-difference criterion.

In conclusion, our approach provides a principled framework for identifying the number of structurally meaningful classes in cryo-EM data sets. By replacing arbitrary thresholds with statistically validated decisions, it enhances the interpretability and reproducibility of 3D classification, offering a promising direction for future developments in cryo-EM heterogeneity analysis.

## Figures and Tables

**Table 1:** Distribution of Ribosembly ground-truth classes (shown as a percentage of the total number of images) and their assignment to selected classification outputs after multiple rounds of classification Classes C1 and C2 follow the first statistically reliable classification. The numbers represent the percentage of the underlying true class images in each of the calculated classes. Classes C11, C12, C13 and C14 correspond to the statistically reliable split of class C1 into statistically distinguishable subsets. Similarly, classes C21 and C22 correspond to the statistically reliable split of class C2. The last row shows the purity of each class, which is computed as the maximum value in the column divided by the sum of all the values.

True class	Total (%)	C1	C2	C11	C12	C13	C14	C21	C22
0	2.7	87.1	12.7	15.3	68.0	2.4	1.4	10.0	2.6
1	4.3	93.8	6.1	5.7	87.3	0.5	0.3	4.4	1.7
2	7.0	95.4	4.5	2.4	92.4	0.4	0.2	3.3	1.2
3	13.2	99.2	0.8	98.9	0.1	0.1	0.1	0.6	0.2
4	9.1	99.5	0.5	99.4	0.0	0.0	0.1	0.4	0.1
5	11.5	98.8	1.1	0.7	0.2	97.6	0.4	0.9	0.3
6	1.2	99.7	0.3	15.0	0.0	0.1	83.7	0.3	0.0
7	1.2	22.8	76.9	22.0	0.1	0.3	0.4	75.8	1.1
8	3.8	0.5	99.5	0.3	0.0	0.0	0.2	99.5	0.1
9	3.6	99.9	0.1	0.3	0.0	0.1	99.5	0.1	0.0
10	5.4	99.4	0.6	0.2	0.0	0.1	99.0	0.6	0.0
11	1.5	88.7	11.1	0.6	0.0	0.2	87.8	6.3	4.7
12	10.5	0.1	99.9	0.0	0.0	0.0	0.0	99.9	0.0
13	11.2	0.1	99.9	0.0	0.0	0.0	0.1	99.9	0.0
14	12.1	0.4	99.6	0.1	0.0	0.0	0.2	0.1	99.5
15	1.7	1.5	98.4	0.6	0.0	0.2	0.7	0.3	98.1
Purity		0.10	0.16	0.38	0.37	0.96	0.27	0.25	0.48
